# Tris(2-ethyl-1*H*-imidazole-κ*N* ^3^)(terephthalato-κ*O*)zinc(II)

**DOI:** 10.1107/S1600536809014755

**Published:** 2009-04-25

**Authors:** Quan-An Xie, Gui-Ying Dong, Ya-Mei Yu, Yong-Gang Wang

**Affiliations:** a School of Chemical and Environmental Engineering, China University of Mining and Technology (Beijing), Beijing 100083, People’s Republic of China; b College of Chemical Engineering and Biotechnology, Hebei Polytechnic University, Tangshan 063009, People’s Republic of China

## Abstract

The title compound, [Zn(C_8_H_4_O_4_)(C_5_H_8_N_2_)_3_], has a neutral monomeric structure in which one terephthalate dianion and three 2-ethyl-1*H*-imidazole ligands coordinate to the Zn^II^ ion in a distorted tetra­hedral geometry. The methyl group of one of the ethyl groups is disordered over two positions with occupancies of 0.66 (2) and 0.34 (2). In the crystal structure, mol­ecules are linked into a three-dimensional hydrogen-bonded network by inter­molecular N—H⋯O interactions involving the uncoordinated carboxyl­ate O atoms.

## Related literature

For the crystal structures of related Zn^II^ complexes, see: Chen *et al.* (1994 ▶); Kimura *et al.* (1991 ▶); Yang *et al.* (2002 ▶).
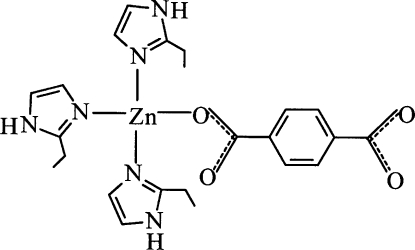


## Experimental

### 

#### Crystal data

[Zn(C_8_H_4_O_4_)(C_5_H_8_N_2_)_3_]
*M* *_r_* = 517.90
Monoclinic, 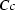

*a* = 11.548 (2) Å
*b* = 11.759 (2) Å
*c* = 18.719 (4) Å
β = 91.79 (3)°
*V* = 2540.7 (8) Å^3^
*Z* = 4
Mo *K*α radiation
μ = 1.01 mm^−1^
*T* = 293 K
0.30 × 0.25 × 0.22 mm

#### Data collection

Bruker SMART CCD area-detector diffractometer
Absorption correction: multi-scan (*SADABS*; Sheldrick, 1996 ▶) *T*_min_ = 0.742, *T*_max_ = 0.812
13021 measured reflections
5719 independent reflections
3534 reflections with *I* > 2σ(*I*)
*R*_int_ = 0.086

#### Refinement

*R*[*F* ^2^ > 2σ(*F* ^2^)] = 0.073
*wR*(*F* ^2^) = 0.114
*S* = 1.12
5719 reflections
317 parameters
16 restraints
H-atom parameters constrained
Δρ_max_ = 0.51 e Å^−3^
Δρ_min_ = −0.28 e Å^−3^
Absolute structure: Flack (1983 ▶), 2826 Friedel pairs
Flack parameter: 0.049 (15)

### 

Data collection: *SMART* (Bruker, 1998 ▶); cell refinement: *SAINT* (Bruker, 1999 ▶); data reduction: *SAINT*; program(s) used to solve structure: *SHELXS97* (Sheldrick, 2008 ▶); program(s) used to refine structure: *SHELXL97* (Sheldrick, 2008 ▶); molecular graphics: *SHELXTL* (Sheldrick, 2008 ▶); software used to prepare material for publication: *SHELXTL*.

## Supplementary Material

Crystal structure: contains datablocks I, global. DOI: 10.1107/S1600536809014755/ci2775sup1.cif

Structure factors: contains datablocks I. DOI: 10.1107/S1600536809014755/ci2775Isup2.hkl

Additional supplementary materials:  crystallographic information; 3D view; checkCIF report

## Figures and Tables

**Table 1:** Selected bond lengths (Å)

Zn1—O1	1.947 (4)
Zn1—N3	2.018 (4)
Zn1—N5	2.023 (5)
Zn1—N1	2.044 (5)

**Table 2:** Hydrogen-bond geometry (Å, °)

*D*—H⋯*A*	*D*—H	H⋯*A*	*D*⋯*A*	*D*—H⋯*A*
N2—H2⋯O4^i^	0.86	1.88	2.717 (7)	165
N4—H4⋯O3^ii^	0.86	1.94	2.787 (7)	167
N6—H6⋯O3^iii^	0.86	1.96	2.797 (7)	163

Symmetry codes: (i) 
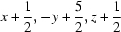
; (ii) 
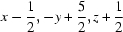
; (iii) 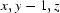.

**Fig. 1.**
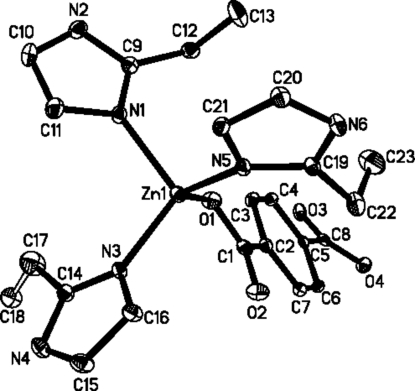
The molecular structure of the title compound, showing the atomic numbering and 30% probability displacement ellipsoids. For clarity, H atoms have been omitted. Only the major disorder component is shown.

## supplementary crystallographic information

### Comment

Metal complexes with imidazole can serve as biomimetic ligands for histidine residues which frequently participate in the co-ordination spheres of metalloenzyme active sites. In particular, carboxylate-histidine-zinc triad systems are regularly observed, and play important roles in the catalytic processes of more than thirty zinc enzymes (Chen *et al.*, 1994). However, crystal structure reports of such model zinc complexes containing neutral imidazole ligands are rather rare, and so far only a few examples have been presented (Kimura *et al.*, 1991; Chen *et al.*, 1994). Here, we report the synthesis and crystal structure of the title complex.

The title compound is a monomeric zinc(II) complex (Fig. 1). The Zn^II^ center is four coordinated by three monodentate 2-ethyl-1*H*-imidazole ligands and by a monodentate terephthalate group, forming a distorted tetrahedral N3,O geometry. The Zn—N bond lengths are in the range 2.014 (6)–2.047 (7) Å and the Zn—O distance is 1.943 (6) Å (Table 1). The most distorted bond angle is O1—Zn1—N1 at 100.5 (3)°.

The N—H···O hydrogen bonds (Table 2) formed between three uncoordinated 2-ethyl-*H*-imidazole N atoms and two uncoordinated carboxylate O atoms, resulted in a three-dimensional hydrogen-bonded network.

### Experimental

The title compound was synthesized by a solvothermal method from Zn(NO_3_)_2_.6H_2_O (29.8 mg, 0.1 mmol), terephthalic acid (77.6 mg,0.4 mmol), 2-ethylimidazole (38.4 mg, 0.4 mmol) and water-ethanol mixed slovent (3 ml). The starting mixture was homogenized and transferred to a sealed Teflon-lined solvothermal bomb (bomb volume: 25 ml) and heated at 433 K for 3 d under autogenous pressure. After cooling in a water bath, colourless crystals were obtained, which were washed and rinsed with distilled water and absolute ethyl alcohol (yield: 51.8% on the basis of Zn(NO_3_)_2_.6H_2_O). Analysis calculated (%) for C_23_H_28_N_6_O_4_Zn: C 53.34, H 5.45, N 16.23%; found: C 53.18, H 5.43, N 16.13.

### Refinement

The methyl C atom, C18, in one of the ethyl groups is disordered over two positions (C18A and C18B) with refined occupancies of 0.66 (2) and 0.34 (2). The C17—C18A and C17—C18B distances were restrained to 1.53 (1) Å. The displacement parameters of the disordered C atoms were also restrained to be approximately isotropic. The aromatic [C-H = 0.93 Å and *U*_iso_(H) = 1.2*U*_eq_(C)] and methylene H atoms [C-H = 0.96 Å and *U*_iso_(H) = 1.5*U*_eq_(C)] were included in the refinement in the riding-model approximation.

### Crystal data

**Table:**

[Zn(C_8_H_4_O_4_)(C_5_H_8_N_2_)_3_]	*F*(000) = 1080
*M**_r_* = 517.90	*D*_x_ = 1.354 Mg m^−^^3^
Monoclinic, *C**c*	Mo *K*α radiation, λ = 0.71073 Å
Hall symbol: C -2yc	Cell parameters from 1240 reflections
*a* = 11.548 (2) Å	θ = 4.5–25.0°
*b* = 11.759 (2) Å	µ = 1.01 mm^−^^1^
*c* = 18.719 (4) Å	*T* = 293 K
β = 91.79 (3)°	Block, white
*V* = 2540.7 (8) Å^3^	0.30 × 0.25 × 0.22 mm
*Z* = 4	

### Data collection

**Table:**

Bruker SMART CCD area-detector diffractometer	5719 independent reflections
Radiation source: fine-focus sealed tube	3534 reflections with *I* > 2σ(*I*)
graphite	*R*_int_ = 0.086
φ and ω scans	θ_max_ = 27.5°, θ_min_ = 3.3°
Absorption correction: multi-scan (*SADABS*; Sheldrick, 1996)	*h* = −14→14
*T*_min_ = 0.742, *T*_max_ = 0.812	*k* = −15→15
13021 measured reflections	*l* = −24→24

### Refinement

**Table:**

Refinement on *F*^2^	Secondary atom site location: difference Fourier map
Least-squares matrix: full	Hydrogen site location: inferred from neighbouring sites
*R*[*F*^2^ > 2σ(*F*^2^)] = 0.073	H-atom parameters constrained
*wR*(*F*^2^) = 0.114	*w* = 1/[σ^2^(*F*_o_^2^) + (0.0189*P*)^2^ + 0.8316*P*] where *P* = (*F*_o_^2^ + 2*F*_c_^2^)/3
*S* = 1.12	(Δ/σ)_max_ = 0.001
5719 reflections	Δρ_max_ = 0.51 e Å^−^^3^
317 parameters	Δρ_min_ = −0.28 e Å^−^^3^
16 restraints	Absolute structure: Flack (1983), 2826 Friedel pairs
Primary atom site location: structure-invariant direct methods	Flack parameter: 0.049 (15)

### Special details

**Table:**

Geometry. All e.s.d.'s (except the e.s.d. in the dihedral angle between two l.s. planes) are estimated using the full covariance matrix. The cell e.s.d.'s are taken into account individually in the estimation of e.s.d.'s in distances, angles and torsion angles; correlations between e.s.d.'s in cell parameters are only used when they are defined by crystal symmetry. An approximate (isotropic) treatment of cell e.s.d.'s is used for estimating e.s.d.'s involving l.s. planes.
Refinement. Refinement of *F*^2^ against ALL reflections. The weighted *R*-factor *wR* and goodness of fit *S* are based on *F*^2^, conventional *R*-factors *R* are based on *F*, with *F* set to zero for negative *F*^2^. The threshold expression of *F*^2^ > σ(*F*^2^) is used only for calculating *R*-factors(gt) *etc*. and is not relevant to the choice of reflections for refinement. *R*-factors based on *F*^2^ are statistically about twice as large as those based on *F*, and *R*- factors based on ALL data will be even larger.

### Fractional atomic coordinates and isotropic or equivalent isotropic displacement parameters (Å^2^)

**Table:**

	*x*	*y*	*z*	*U*_iso_*/*U*_eq_	Occ. (<1)
Zn1	0.31207 (5)	0.87464 (5)	1.00368 (4)	0.04221 (18)	
O1	0.3327 (4)	1.0107 (4)	0.9463 (2)	0.0616 (12)	
O2	0.1668 (4)	0.9779 (4)	0.8862 (3)	0.0651 (13)	
O3	0.4207 (4)	1.5052 (4)	0.7476 (2)	0.0570 (12)	
O4	0.3054 (4)	1.4345 (4)	0.6625 (2)	0.0628 (12)	
N1	0.4459 (4)	0.8920 (4)	1.0769 (2)	0.0459 (13)	
N2	0.6080 (5)	0.9487 (4)	1.1293 (3)	0.0647 (15)	
H2	0.6768	0.9759	1.1355	0.078*	
N3	0.1653 (4)	0.8651 (4)	1.0592 (2)	0.0421 (11)	
N4	0.0232 (5)	0.8949 (5)	1.1303 (3)	0.0639 (15)	
H4	−0.0184	0.9259	1.1622	0.077*	
N5	0.3423 (4)	0.7220 (4)	0.9580 (2)	0.0436 (12)	
N6	0.4022 (5)	0.5903 (4)	0.8863 (3)	0.0529 (14)	
H6	0.4181	0.5548	0.8477	0.063*	
C1	0.2545 (6)	1.0340 (5)	0.8977 (3)	0.0476 (15)	
C2	0.2797 (5)	1.1396 (5)	0.8544 (3)	0.0392 (14)	
C3	0.3797 (5)	1.2032 (5)	0.8667 (3)	0.0443 (15)	
H3	0.4319	1.1816	0.9030	0.053*	
C4	0.4029 (5)	1.2980 (5)	0.8259 (3)	0.0420 (14)	
H4A	0.4702	1.3396	0.8351	0.050*	
C5	0.3255 (5)	1.3317 (4)	0.7707 (3)	0.0341 (13)	
C6	0.2258 (5)	1.2678 (5)	0.7579 (3)	0.0407 (15)	
H6A	0.1738	1.2885	0.7213	0.049*	
C7	0.2034 (5)	1.1723 (5)	0.8001 (3)	0.0447 (15)	
H7	0.1362	1.1304	0.7913	0.054*	
C8	0.3515 (5)	1.4310 (5)	0.7235 (3)	0.0427 (15)	
C9	0.5509 (6)	0.9366 (5)	1.0669 (3)	0.0470 (15)	
C10	0.5378 (8)	0.9101 (7)	1.1818 (4)	0.088 (3)	
H10	0.5553	0.9084	1.2306	0.106*	
C11	0.4387 (6)	0.8751 (6)	1.1490 (4)	0.074 (2)	
H11	0.3754	0.8444	1.1717	0.088*	
C12	0.6028 (5)	0.9657 (5)	0.9961 (3)	0.0558 (18)	
H12A	0.6636	1.0218	1.0038	0.067*	
H12B	0.5436	0.9993	0.9649	0.067*	
C13	0.6528 (8)	0.8621 (7)	0.9599 (4)	0.101 (3)	
H13A	0.6846	0.8842	0.9152	0.152*	
H13B	0.5927	0.8070	0.9515	0.152*	
H13C	0.7128	0.8297	0.9902	0.152*	
C14	0.1249 (5)	0.9330 (5)	1.1079 (3)	0.0498 (16)	
C15	−0.0025 (6)	0.7984 (6)	1.0934 (4)	0.066 (2)	
H15	−0.0683	0.7535	1.0974	0.080*	
C16	0.0867 (5)	0.7808 (5)	1.0497 (4)	0.0542 (17)	
H16	0.0930	0.7203	1.0181	0.065*	
C17	0.1830 (8)	1.0329 (7)	1.1407 (5)	0.110 (3)	
H17A	0.1455	1.0515	1.1848	0.132*	0.661 (18)
H17B	0.2629	1.0134	1.1526	0.132*	0.661 (18)
H17C	0.2519	1.0460	1.1144	0.132*	0.339 (18)
H17D	0.2080	1.0100	1.1879	0.132*	0.339 (18)
C18A	0.1810 (13)	1.1331 (11)	1.0944 (8)	0.115 (6)	0.661 (18)
H18A	0.2203	1.1948	1.1184	0.172*	0.661 (18)
H18B	0.1022	1.1544	1.0835	0.172*	0.661 (18)
H18C	0.2192	1.1158	1.0509	0.172*	0.661 (18)
C18B	0.1283 (18)	1.1454 (12)	1.1528 (14)	0.079 (9)	0.339 (18)
H18D	0.1837	1.1955	1.1756	0.119*	0.339 (18)
H18E	0.0631	1.1362	1.1828	0.119*	0.339 (18)
H18F	0.1029	1.1772	1.1077	0.119*	0.339 (18)
C19	0.3515 (5)	0.6916 (5)	0.8896 (3)	0.0445 (15)	
C20	0.4250 (6)	0.5515 (5)	0.9545 (3)	0.0547 (17)	
H20	0.4601	0.4832	0.9678	0.066*	
C21	0.3857 (5)	0.6335 (5)	0.9983 (3)	0.0482 (15)	
H21	0.3879	0.6304	1.0480	0.058*	
C22	0.3089 (7)	0.7583 (6)	0.8248 (4)	0.070 (2)	
H22A	0.2804	0.7060	0.7883	0.085*	
H22B	0.2450	0.8067	0.8381	0.085*	
C23	0.4025 (8)	0.8302 (8)	0.7949 (5)	0.124 (4)	
H23A	0.3723	0.8713	0.7541	0.187*	
H23B	0.4653	0.7825	0.7808	0.187*	
H23C	0.4301	0.8831	0.8306	0.187*	

### Atomic displacement parameters (Å^2^)

**Table:**

	*U*^11^	*U*^22^	*U*^33^	*U*^12^	*U*^13^	*U*^23^
Zn1	0.0425 (3)	0.0409 (3)	0.0436 (4)	0.0011 (5)	0.0069 (2)	0.0037 (4)
O1	0.059 (3)	0.059 (3)	0.067 (3)	0.006 (2)	0.004 (2)	0.027 (2)
O2	0.052 (3)	0.052 (3)	0.092 (4)	−0.005 (2)	0.005 (3)	0.017 (3)
O3	0.081 (3)	0.052 (3)	0.039 (3)	−0.003 (3)	0.015 (2)	0.010 (2)
O4	0.066 (3)	0.078 (3)	0.044 (3)	0.007 (3)	−0.005 (2)	0.020 (3)
N1	0.051 (3)	0.050 (3)	0.037 (3)	−0.008 (3)	0.003 (2)	0.003 (2)
N2	0.060 (4)	0.071 (4)	0.062 (4)	−0.004 (3)	−0.012 (3)	−0.011 (3)
N3	0.045 (3)	0.042 (3)	0.040 (3)	0.000 (3)	0.009 (2)	−0.005 (3)
N4	0.063 (4)	0.065 (4)	0.064 (4)	0.009 (3)	0.021 (3)	−0.012 (3)
N5	0.047 (3)	0.050 (3)	0.035 (3)	0.003 (2)	0.005 (2)	0.001 (2)
N6	0.071 (4)	0.043 (3)	0.045 (3)	0.007 (3)	0.015 (3)	0.000 (3)
C1	0.050 (4)	0.042 (4)	0.052 (4)	0.013 (3)	0.011 (3)	0.002 (3)
C2	0.044 (3)	0.035 (3)	0.038 (3)	0.005 (3)	0.004 (3)	0.002 (3)
C3	0.053 (4)	0.040 (4)	0.039 (4)	0.007 (3)	−0.007 (3)	0.007 (3)
C4	0.041 (4)	0.041 (4)	0.043 (4)	0.001 (3)	−0.002 (3)	0.001 (3)
C5	0.038 (3)	0.039 (3)	0.026 (3)	0.010 (3)	0.008 (2)	0.002 (3)
C6	0.037 (4)	0.051 (4)	0.033 (3)	0.009 (3)	−0.006 (3)	−0.002 (3)
C7	0.041 (4)	0.050 (4)	0.043 (4)	0.003 (3)	0.002 (3)	−0.003 (3)
C8	0.041 (4)	0.046 (4)	0.042 (4)	0.013 (3)	0.013 (3)	0.007 (3)
C9	0.058 (4)	0.029 (3)	0.053 (4)	0.003 (3)	0.000 (3)	−0.006 (3)
C10	0.095 (7)	0.127 (8)	0.042 (5)	0.006 (6)	−0.003 (5)	−0.008 (5)
C11	0.071 (5)	0.096 (6)	0.053 (5)	−0.004 (5)	0.002 (4)	0.013 (4)
C12	0.042 (4)	0.062 (5)	0.064 (5)	−0.010 (3)	0.005 (3)	0.004 (4)
C13	0.110 (7)	0.102 (7)	0.095 (7)	−0.010 (6)	0.047 (5)	−0.036 (6)
C14	0.049 (4)	0.049 (4)	0.052 (4)	−0.005 (3)	0.004 (3)	−0.010 (3)
C15	0.049 (4)	0.060 (5)	0.091 (6)	−0.016 (4)	0.011 (4)	−0.008 (4)
C16	0.044 (4)	0.046 (4)	0.073 (5)	−0.007 (3)	0.008 (3)	−0.019 (3)
C17	0.105 (8)	0.092 (7)	0.134 (8)	−0.003 (6)	0.015 (6)	−0.032 (6)
C18A	0.116 (9)	0.103 (9)	0.125 (10)	−0.004 (7)	0.002 (7)	0.009 (7)
C18B	0.077 (11)	0.069 (12)	0.092 (12)	−0.004 (8)	0.006 (8)	−0.009 (8)
C19	0.051 (4)	0.042 (4)	0.041 (4)	0.000 (3)	0.009 (3)	0.002 (3)
C20	0.066 (4)	0.045 (4)	0.053 (4)	0.009 (3)	0.007 (3)	0.005 (3)
C21	0.052 (4)	0.055 (4)	0.038 (3)	−0.001 (3)	−0.001 (3)	0.002 (3)
C22	0.101 (6)	0.056 (5)	0.054 (5)	0.020 (4)	0.012 (4)	0.006 (4)
C23	0.119 (8)	0.167 (10)	0.089 (7)	0.057 (7)	0.028 (6)	0.054 (7)

### Geometric parameters (Å, °)

**Table:**

Zn1—O1	1.947 (4)	C10—C11	1.346 (10)
Zn1—N3	2.018 (4)	C10—H10	0.93
Zn1—N5	2.023 (5)	C11—H11	0.93
Zn1—N1	2.044 (5)	C12—C13	1.516 (9)
O1—C1	1.291 (7)	C12—H12A	0.97
O2—C1	1.223 (7)	C12—H12B	0.97
O3—C8	1.257 (7)	C13—H13A	0.96
O4—C8	1.245 (7)	C13—H13B	0.96
N1—C9	1.339 (7)	C13—H13C	0.96
N1—C11	1.370 (8)	C14—C17	1.477 (9)
N2—C9	1.331 (8)	C15—C16	1.351 (8)
N2—C10	1.371 (9)	C15—H15	0.93
N2—H2	0.86	C16—H16	0.93
N3—C14	1.309 (7)	C17—C18A	1.462 (8)
N3—C16	1.352 (7)	C17—C18B	1.486 (9)
N4—C14	1.336 (7)	C17—H17A	0.97
N4—C15	1.356 (8)	C17—H17B	0.97
N4—H4	0.86	C17—H17C	0.96
N5—C19	1.337 (7)	C17—H17D	0.96
N5—C21	1.371 (7)	C18A—H18A	0.96
N6—C19	1.330 (7)	C18A—H18B	0.96
N6—C20	1.373 (7)	C18A—H18C	0.96
N6—H6	0.86	C18B—H18D	0.96
C1—C2	1.516 (8)	C18B—H18E	0.96
C2—C7	1.379 (7)	C18B—H18F	0.96
C2—C3	1.389 (8)	C19—C22	1.513 (9)
C3—C4	1.382 (7)	C20—C21	1.354 (7)
C3—H3	0.93	C20—H20	0.93
C4—C5	1.402 (7)	C21—H21	0.93
C4—H4A	0.93	C22—C23	1.495 (11)
C5—C6	1.389 (7)	C22—H22A	0.97
C5—C8	1.501 (7)	C22—H22B	0.97
C6—C7	1.401 (8)	C23—H23A	0.96
C6—H6A	0.93	C23—H23B	0.96
C7—H7	0.93	C23—H23C	0.96
C9—C12	1.512 (8)		
			
O1—Zn1—N3	116.67 (18)	C13—C12—H12B	109.2
O1—Zn1—N5	118.06 (18)	H12A—C12—H12B	107.9
N3—Zn1—N5	109.09 (19)	C12—C13—H13A	109.5
O1—Zn1—N1	100.7 (2)	C12—C13—H13B	109.5
N3—Zn1—N1	106.87 (18)	H13A—C13—H13B	109.5
N5—Zn1—N1	103.6 (2)	C12—C13—H13C	109.5
C1—O1—Zn1	117.9 (4)	H13A—C13—H13C	109.5
C9—N1—C11	106.1 (5)	H13B—C13—H13C	109.5
C9—N1—Zn1	127.9 (4)	N3—C14—N4	110.5 (5)
C11—N1—Zn1	125.3 (4)	N3—C14—C17	127.4 (6)
C9—N2—C10	107.8 (6)	N4—C14—C17	121.9 (6)
C9—N2—H2	126.1	C16—C15—N4	106.1 (6)
C10—N2—H2	126.1	C16—C15—H15	127.0
C14—N3—C16	106.6 (5)	N4—C15—H15	127.0
C14—N3—Zn1	130.4 (4)	C15—C16—N3	109.3 (6)
C16—N3—Zn1	123.0 (4)	C15—C16—H16	125.4
C14—N4—C15	107.6 (5)	N3—C16—H16	125.4
C14—N4—H4	126.2	C18A—C17—C14	113.4 (9)
C15—N4—H4	126.2	C14—C17—C18B	125.6 (11)
C19—N5—C21	106.6 (5)	C18A—C17—H17A	108.9
C19—N5—Zn1	131.5 (4)	C14—C17—H17A	108.9
C21—N5—Zn1	120.4 (4)	C18A—C17—H17B	108.9
C19—N6—C20	109.1 (5)	C14—C17—H17B	108.9
C19—N6—H6	125.4	H17A—C17—H17B	107.7
C20—N6—H6	125.4	C14—C17—H17C	106.7
O2—C1—O1	124.6 (6)	C18B—C17—H17C	107.3
O2—C1—C2	121.3 (6)	C14—C17—H17D	106.2
O1—C1—C2	114.1 (6)	C18B—C17—H17D	103.1
C7—C2—C3	118.7 (5)	H17C—C17—H17D	106.7
C7—C2—C1	119.6 (5)	C17—C18A—H18A	109.5
C3—C2—C1	121.7 (5)	C17—C18A—H18B	109.5
C4—C3—C2	121.2 (5)	H18A—C18A—H18B	109.5
C4—C3—H3	119.4	C17—C18A—H18C	109.5
C2—C3—H3	119.4	H18A—C18A—H18C	109.5
C3—C4—C5	120.3 (5)	H18B—C18A—H18C	109.5
C3—C4—H4A	119.8	C17—C18B—H18D	109.5
C5—C4—H4A	119.8	C17—C18B—H18E	109.5
C6—C5—C4	118.7 (5)	H18D—C18B—H18E	109.5
C6—C5—C8	120.0 (5)	C17—C18B—H18F	109.5
C4—C5—C8	121.3 (5)	H18D—C18B—H18F	109.5
C5—C6—C7	120.2 (5)	H18E—C18B—H18F	109.5
C5—C6—H6A	119.9	N6—C19—N5	109.4 (5)
C7—C6—H6A	119.9	N6—C19—C22	124.1 (6)
C2—C7—C6	120.9 (6)	N5—C19—C22	126.5 (6)
C2—C7—H7	119.5	C21—C20—N6	105.6 (5)
C6—C7—H7	119.5	C21—C20—H20	127.2
O4—C8—O3	123.7 (6)	N6—C20—H20	127.2
O4—C8—C5	118.5 (6)	C20—C21—N5	109.3 (5)
O3—C8—C5	117.7 (5)	C20—C21—H21	125.3
N2—C9—N1	110.3 (6)	N5—C21—H21	125.3
N2—C9—C12	123.0 (6)	C23—C22—C19	111.9 (7)
N1—C9—C12	126.7 (6)	C23—C22—H22A	109.2
C11—C10—N2	106.8 (7)	C19—C22—H22A	109.2
C11—C10—H10	126.6	C23—C22—H22B	109.2
N2—C10—H10	126.6	C19—C22—H22B	109.2
C10—C11—N1	109.1 (7)	H22A—C22—H22B	107.9
C10—C11—H11	125.5	C22—C23—H23A	109.5
N1—C11—H11	125.5	C22—C23—H23B	109.5
C9—C12—C13	112.1 (5)	H23A—C23—H23B	109.5
C9—C12—H12A	109.2	C22—C23—H23C	109.5
C13—C12—H12A	109.2	H23A—C23—H23C	109.5
C9—C12—H12B	109.2	H23B—C23—H23C	109.5
			
N3—Zn1—O1—C1	−58.2 (5)	C4—C5—C8—O3	−23.6 (8)
N5—Zn1—O1—C1	74.8 (4)	C10—N2—C9—N1	0.3 (7)
N1—Zn1—O1—C1	−173.4 (4)	C10—N2—C9—C12	−177.4 (6)
O1—Zn1—N1—C9	−34.4 (5)	C11—N1—C9—N2	−0.5 (7)
N3—Zn1—N1—C9	−156.7 (5)	Zn1—N1—C9—N2	170.7 (4)
N5—Zn1—N1—C9	88.1 (5)	C11—N1—C9—C12	177.1 (6)
O1—Zn1—N1—C11	135.2 (5)	Zn1—N1—C9—C12	−11.7 (9)
N3—Zn1—N1—C11	12.9 (6)	C9—N2—C10—C11	0.1 (8)
N5—Zn1—N1—C11	−102.3 (6)	N2—C10—C11—N1	−0.3 (9)
O1—Zn1—N3—C14	−58.6 (6)	C9—N1—C11—C10	0.5 (8)
N5—Zn1—N3—C14	164.5 (5)	Zn1—N1—C11—C10	−171.0 (5)
N1—Zn1—N3—C14	53.0 (6)	N2—C9—C12—C13	96.4 (7)
O1—Zn1—N3—C16	120.4 (5)	N1—C9—C12—C13	−80.8 (8)
N5—Zn1—N3—C16	−16.6 (5)	C16—N3—C14—N4	0.2 (7)
N1—Zn1—N3—C16	−128.0 (5)	Zn1—N3—C14—N4	179.3 (4)
O1—Zn1—N5—C19	−16.2 (6)	C16—N3—C14—C17	175.1 (7)
N3—Zn1—N5—C19	120.0 (5)	Zn1—N3—C14—C17	−5.7 (10)
N1—Zn1—N5—C19	−126.4 (5)	C15—N4—C14—N3	−0.4 (7)
O1—Zn1—N5—C21	147.3 (4)	C15—N4—C14—C17	−175.7 (7)
N3—Zn1—N5—C21	−76.4 (4)	C14—N4—C15—C16	0.5 (8)
N1—Zn1—N5—C21	37.2 (5)	N4—C15—C16—N3	−0.4 (8)
Zn1—O1—C1—O2	1.8 (8)	C14—N3—C16—C15	0.1 (8)
Zn1—O1—C1—C2	−178.4 (3)	Zn1—N3—C16—C15	−179.1 (5)
O2—C1—C2—C7	−1.2 (8)	N3—C14—C17—C18A	77.0 (12)
O1—C1—C2—C7	179.0 (5)	N4—C14—C17—C18A	−108.6 (11)
O2—C1—C2—C3	−179.4 (5)	N3—C14—C17—C18B	135.1 (15)
O1—C1—C2—C3	0.8 (8)	N4—C14—C17—C18B	−50.5 (17)
C7—C2—C3—C4	0.4 (8)	C20—N6—C19—N5	1.3 (7)
C1—C2—C3—C4	178.6 (5)	C20—N6—C19—C22	−177.2 (6)
C2—C3—C4—C5	−0.3 (8)	C21—N5—C19—N6	−2.1 (6)
C3—C4—C5—C6	−0.2 (8)	Zn1—N5—C19—N6	163.1 (4)
C3—C4—C5—C8	−177.1 (5)	C21—N5—C19—C22	176.4 (6)
C4—C5—C6—C7	0.5 (8)	Zn1—N5—C19—C22	−18.4 (9)
C8—C5—C6—C7	177.5 (5)	C19—N6—C20—C21	0.1 (7)
C3—C2—C7—C6	0.0 (8)	N6—C20—C21—N5	−1.4 (7)
C1—C2—C7—C6	−178.3 (5)	C19—N5—C21—C20	2.2 (7)
C5—C6—C7—C2	−0.4 (8)	Zn1—N5—C21—C20	−165.0 (4)
C6—C5—C8—O4	−21.7 (8)	N6—C19—C22—C23	−86.7 (9)
C4—C5—C8—O4	155.2 (5)	N5—C19—C22—C23	95.0 (8)
C6—C5—C8—O3	159.5 (5)		

### Hydrogen-bond geometry (Å, °)

**Table:**

*D*—H···*A*	*D*—H	H···*A*	*D*···*A*	*D*—H···*A*
N2—H2···O4^i^	0.86	1.88	2.717 (7)	165
N4—H4···O3^ii^	0.86	1.94	2.787 (7)	167
N6—H6···O3^iii^	0.86	1.96	2.797 (7)	163

Symmetry codes: (i) *x*+1/2, −*y*+5/2, *z*+1/2; (ii) *x*−1/2, −*y*+5/2, *z*+1/2; (iii) *x*, *y*−1, *z*.
